# The Study of Cellular and Molecular Physiological Characteristics of Sperm in Men Living in the Aral Sea Region

**DOI:** 10.3889/oamjms.2016.007

**Published:** 2015-12-22

**Authors:** Berikbay Z. Kultanov, Raushan S. Dosmagambetova, Svetlana A. Ivasenko, Yelena S. Tatina, Assel A. Kelmyalene, Lyazzat H. Assenova

**Affiliations:** *Karaganda State Medical University, Department of the Republic of Kazakhstan, Gogolya street 40, Karaganda, Kazakhstan*

**Keywords:** Aral Sea, ASF, RNA, DNA, H1, H2A, H3, H4

## Abstract

**BACKGROUND:**

Extreme environmental situation in the Aral crisis has caused a massive chemical pollution of the territory for decades with high doses of pesticides, herbicides. Discharge of industrial waste into the rivers that feed the Aral Sea has lead to the development of various pathological processes in the human body, as well as disruption of reproductive function in young men.

**AIM:**

To evaluate the performance of molecular cellular changes in the sperm of men under the influence of dust and salt aerosols in Aral Sea region.

**MATERIAL AND METHODS:**

Clinical and laboratory studies were conducted in men 5 settlements (Aralsk-city, v. Aiteke-Bi, v. Zhalagash, v. Zhusaly, v. Shieli). We have studied male ejaculate obtained after 4-5 days of abstinence, and placed it in a warm tube with a glass stopper. On the investigation proceeded ejaculate within 20-30 minutes after its preparation, during which time he was subjected to liquefaction. Isolation and quantification of ASF, RNA, DNA, and determining the fraction of histones in sperm was performed by the method of Markusheva and Savina.

**RESULTS:**

It was found that the value of ASF in the semen of men living in the zone of ecological disaster higher compared with the values of parameters in men living in the area of environmental crisis, and this trend is observed in all age groups. The study of circulating extracellular DNA and RNA in the sperm of men registered their decline with a corresponding increase of acid precursors that can be attributed to the degradation of nucleic acids under the influence of negative factors in the complex area of ecological trouble. Also, according to a study in men residing in the areas of environmental catastrophe at the age of 18-29 years, found an increased content of the H1 histone H2A lower total fraction, H3, H4 - and a sharp increase in histone H2B content - histones.

**CONCLUSIONS:**

Men living in environmentally disadvantaged areas of Kyzylorda region under the influence of dust and salt aerosols and other toxicants leads to disruption of the reproductive function in men.

## Introduction

The Aral Sea crisis is recognized as one of the global environmental problems of our time. Extreme ecological situation in the Aral crisis caused a massive chemical pollution of the territory for decades with high doses of pesticides, herbicides; discharge of industrial waste into the rivers that feed the Aral Sea. Due to the drying of the Aral Sea occurred arid areas, climate change and soil salinization, breach of public water supply.

Existing environmental trouble in the region is reflected in the health of the population in almost all areas of the Aral Sea region is marked increase in the number of diseases of the endocrine, nervous, digestive and urinary systems [1]. Numerous studies conducted by scientists of Kazakhstan shows that the state of health of the population in recent decades, the Aral Sea region continues to deteriorate [2].

Previous studies on a range of health, social and environmental problems of the Aral Sea region is mainly devoted to the study of the sanitary-epidemiological situation and the health of the population only in the zone of ecological disaster - the Aral and Kazalinsk areas. Most research in this area relate to aspects of child health and reproductive health of women [3-5].

The problem of male infertility in recent years is of particular medical and social importance due to the increased incidence of sexual organs, increases in congenital malformations caused by environmental pollution, extensive and uncontrolled use of drugs [6-8] and has not been studied in men of reproductive age living in the area ecological catastrophes and ecological crisis of Kyzylorda region.

Therefore, the purpose of work is to study the negative impact of environmental factors on the stages of spermatogenesis in men of reproductive age living in the zone of ecological disaster and environmental crisis Kyzylorda region.

## Material and Methods

The studies were conducted in the Laboratory of the Department of Molecular Biology and Medical Genetics, KSMU.

Clinical and laboratory studies were conducted in men 5 settlements (Aralsk-city, v. Aiteke-Bi, v. Zhalagash, v. Zhusaly, v. Shieli). They were surveyed 1,010 people the same age group (18-49 years). The criterion for inclusion is the stay of an adult in the Aral Sea area is not less than 5 years, employment in occupations with the hazard no more than 2 classes.

We have studied male ejaculate obtained after 4-5 days of abstinence, and placed it in a warm tube with a glass stopper. On the investigation proceeded ejaculate within 20-30 minutes after its preparation, during which time he was subjected to liquefaction. Macroscopic and microscopic examination of the semen was performed by the method of V. Dolgov. Isolation and quantification of ASF, RNA, DNA, and determining the fraction of histones in sperm was performed by the method of L. Markusheva and M.I. Savina [7].

Morphological studies of ejaculate of surveyed persons living in the Aral Sea region held on a light microscope MICROS model MC 20 equipped with a digital camera eyepiece OSMOSIS 14000KPA. Molecular and cellular studies were performed on a digital spectrophotometer UV-VIS PD-303UV.

## Results

Freshly ejaculate initially evaluated by external characteristics, our findings showed that, in all the surveyed men living in the areas of environmental disasters of Kyzylorda region, there are changes in the integral characteristics of the ejaculate. So, almost all the investigated areas there is a high percentage of individuals with a transparent color ejaculate in all age groups (21 to 42%), indicating a reduced amount of sperm in the ejaculate.

Ejaculate healthy male should have a characteristic odor; no smell indicates a lack of secretion of the prostate. The presence or absence of odor was observed in the surveyed people in all 5 areas. The maximum number of persons to the lack of odor ejaculate was noted in Aiteke Bi in Aralsk (37.4% and 41.7%).

Also, the persons surveyed all 5 groups observed some increased ability to ejaculate liquefaction, according to the WHO the duration of thinning in men ejaculate normally ranges from 15 to 30 minutes, averaging 29.7 minutes. The highest percentage of people over time, liquefaction of the ejaculate to 2 minutes was observed in living in Aralsk (61.6%) and up to 5 minutes in Aiteke-Bi (39.2%), among the highest number of men with liquefaction time less than 15 minutes noted in Zhusaly (36%) (Table 1).

**Table 1:** Time ejaculate liquefaction of the surveyed persons

Groups		Liquefaction time. min			
		2 min	5 min	15 min	15-30 min
18-29 years old					
Norm According to the World Health Organization		-	-		100
v. Ayteke Bi		48.3%	16.7%	35%	-
Aralsk- city		52.5%	17%	30.5%	-
v. Zhusaly		35%	28%	37%	-
v. Zhalagash		27.4%	28.6%	42%	-
v. Shieli		38.2%	30.8%	31%	-
30-39 years old					
Norm According to the World Health Organization		-	-		100
v. Ayteke Bi		30.2%	59.8%	10%	-
Aralsk- city		49.6%	24.4%	26%	-
v. Zhusaly		33%	18%	49%	-
v. Zhalagash		26%	51.3%	22.7%	-
v. Shieli		33.6%	29.4%	37%	-
40-49 years old					
Norm According to the World Health Organization		-	-		100
v. Ayteke Bi		41.3%	39.2%	19.5%	-
Aralsk- city		61.6%	25.2%	13.2%	-
v. Zhusaly		35.7%	28.3%	36%	-
v. Zhalagash		37.1%	29.9%	33%	-
v. Shieli		49.2%	26.4%	24.4%	-

According to the regulations, sperm count below 60 million in 1 ml shows a decrease in fertility ejaculate. According to the literature found that healthy male sperm count in 1 ml of ejaculate varies between 70 million in 1 ml and above. In all five groups surveyed persons of all ages observed low sperm count, that is, less than 70 million in 1 ml of ejaculate. A comparative analysis of not prolific ejaculate had been done in all age groups of surveyed men. It was found that relatively high values of not prolific semen found in the age group 18-29 years in all five districts of Kyzylorda region, the highest percentage observed in the zone of ecological disaster - in Aralsk (84.7%).

In addition, from the results of research showed the maximum increase in the number of mobile forms of sperm in men v. Shieli, the maximum amount of sperm ejaculate with reduced fertility observed in v. Zhusaly and with significantly reduced fertility in v. Aiteke Bi. Fixed forms of spermatozoa observed in Aralsk and v. Aiteke Bi.

The study examined all men living in the Kyzylorda region, there are deviations from the norm WHO morphophysiological characteristics of sperm.

In assessing the pathological forms of sperm attach special importance to the changes of the head, which for the fertility of an ejaculate are more important than changes in the intermediate portion and the tail. In the area of environmental disaster (v. Aiteke Bi and c. Aralsk) of the men surveyed, depending on the age of the following changes are observed: deformation of the head and the body of sperm abnormality found in men aged 18 to 29 years (up to 26.0% and 18 %, respectively). When comparing the pathological forms of sperm in men living in the zone of ecological crisis, a high percentage of abnormal deformation of the head and the body of sperm were observed in men aged 18-39 years in villages Zhalagash and Shieli.

Analysis of clinical and laboratory research showed that men living in ecologically unfavorable conditions Kyzylorda region there are persistent violations of spermatogenesis. Of particular note is that a violation of the morphological and physiological characteristics of sperm is most significant in young adults. The picture of these disorders manifested itself in the form of increasing the number of fixed sperm cells, the appearance of atypical forms of the deformation of the head and the doubling of the axoneme.

We performed molecular genetic studies of acid-soluble fractions (ASF), extracellular nucleic acids (RNA, DNA) and histone-similar fractions (H1, H2A, H3, H4) in the ejaculate of men in the zone of ecological disaster Kyzylorda region (Table 2 and 3).

**Table 2:** Indicators of extracellular nucleic acids in semen of men (M ± m)

	years	ASF, conventional units	RNA, conventional units	DNA, conventional units
The zone of ecological disaster (v. Ayteke Bi Aralsk-city)	18-29 years	0.72 ± 0.13	0.73 ± 0.12	0.49 ± 0.058

	30-39 years	0.68 ± 0.087	0.88 ± 0.082	0.57 ± 0.04

	40-49 years	0.63 ± 0.09	0.76 ± 0.094	0.46 ± 0.063

The zone of ecological crisis (v. Zhosaly, v. Zhalagash, v. Shieli)	18-29 years	0.58 ± 0.4	3.13 ± 2.61^**^	0.42 ± 0.034^*^

	30-39 years	0.63 ± 0.05^*^	0.7 ± 0.053^**^	0.56 ± 0.054

	40-49 years	0.46 ± 0.06^*^	0.6 ± 0.048	0.69 ± 0.09^*^

Note: reliability, compared with groups of different ecological zones, p <0.001

** - significant in comparison with groups of different ecological zones, p <0.001

**Table 3:** Indicators of histones in sperm in men (M ± m)

	years	H_1_. ml/l	H_2_A, H_3_, H_4_, ml/sl	H_2_B, ml/l
The zone of ecological disaster (v. Ayteke Bi Aralsk-city)	18-29 years	0.83 ± 0.18^*^	0.81 ± 0.032	1.59 ± 0.084^*^
	30-39 years	0.69 ± 0.062	0.51 ± 0.059^*^	0.83 ± 0.07^*^
	40-49 years	0.74 ± 0.036	0.71 ± 0.091	1.062 ± 0.074
The zone of ecological crisis (v. Zhosaly, v. Zhalagash, v. Shieli)	18-29 years	0.60 ± 0.035	0.61 ± 0.030	0.61 ± 0.029
	30-39 years	0.54 ± 0.046	0.61 ± 0.039	0.66 ± 0.039
	40-49 years	0.56 ± 0.050	0.68 ± 0.036	0.72 ± 0.046

Note: - reliability, compared with groups of different ecological zones, p <0.001, ** - significant in comparison with groups of different ecological zones, p <0.001

It was found that the value of ASF in the semen of men living in the zone of ecological disaster higher compared with the values of parameters in men living in the area of environmental crisis, and this trend is observed in all age groups. The study of circulating extracellular DNA and RNA in the sperm of men registered their decline with a corresponding increase of acid precursors that can be attributed to the degradation of nucleic acids under the influence of negative factors in the complex area of ecological trouble. Also, according to a study in men residing in the areas of environmental catastrophe at the age of 18-29 years, found an increased content of the H1 histone H2A lower total fraction, H3, H4 - and a sharp increase in histone H2B content - histones.

Men living in zones of ecological crisis of all age groups showed an increased content of total fraction of H2A, H3, H4 and H2B histones.

## Discussion

When comparing the results of the men living in the zones of ecological disaster and environmental crisis in all age groups may be noted the high content of H1 histone-similar factions in individuals living in v. Zhusaly, v. Zhalagash and v. Shieli.

According to a study in the sperm of men who live in the zones of ecological disaster, there is a different dynamics of the studied parameters in different areas, which determines the extent of metabolic abnormalities in sperm.

Thus, the violation of morphological parameters of spermatogenesis was observed in all the surveyed people in the zone of ecological crisis and environmental disasters. These changes lead to the development of pathological processes at the molecular and cellular level. This indicates a change of extracellular nucleic acids and histones in the semen of the surveyed men living in environmentally disadvantaged areas of Kyzylorda region. In this regard, we can assume that the dust and salt aerosols and ecotoxicants adversely affect the reproductive health of the local male population. Our assumption is confirmed by the results of other domestic researchers. In recent years, the world in general and in particular in the Aral Sea region, much attention is paid to the impact on the human body of heavy metals, especially lead [9]. The destruction of natural ecosystems, degradation of flora and fauna and as a consequence of the unfavorable ecological situation caused substantial harm to the health of the population [10]. The deterioration of the environment impact on health status [2].

Pathology affects the reproductive system disorders of the immune system and the biochemical, pathophysiological changes.

The problem of male infertility in recent years is of particular medical and social importance all over the world.
